# Supplementary material for: Physiological skin FDG uptake: A quantitative and regional distribution assessment using PET/MRI
Source: PLoS One. 2021 Mar 26;16(3):e0249304. doi: 10.1371/journal.pone.0249304 (PMC7997016; doi:10.1371/journal.pone.0249304)
Supplement: S1 Fig — The graphs show face (A), scalp (B), chest (C), abdomen (D), and back (E) regions. (DOCX) [file pone.0249304.s001.docx]

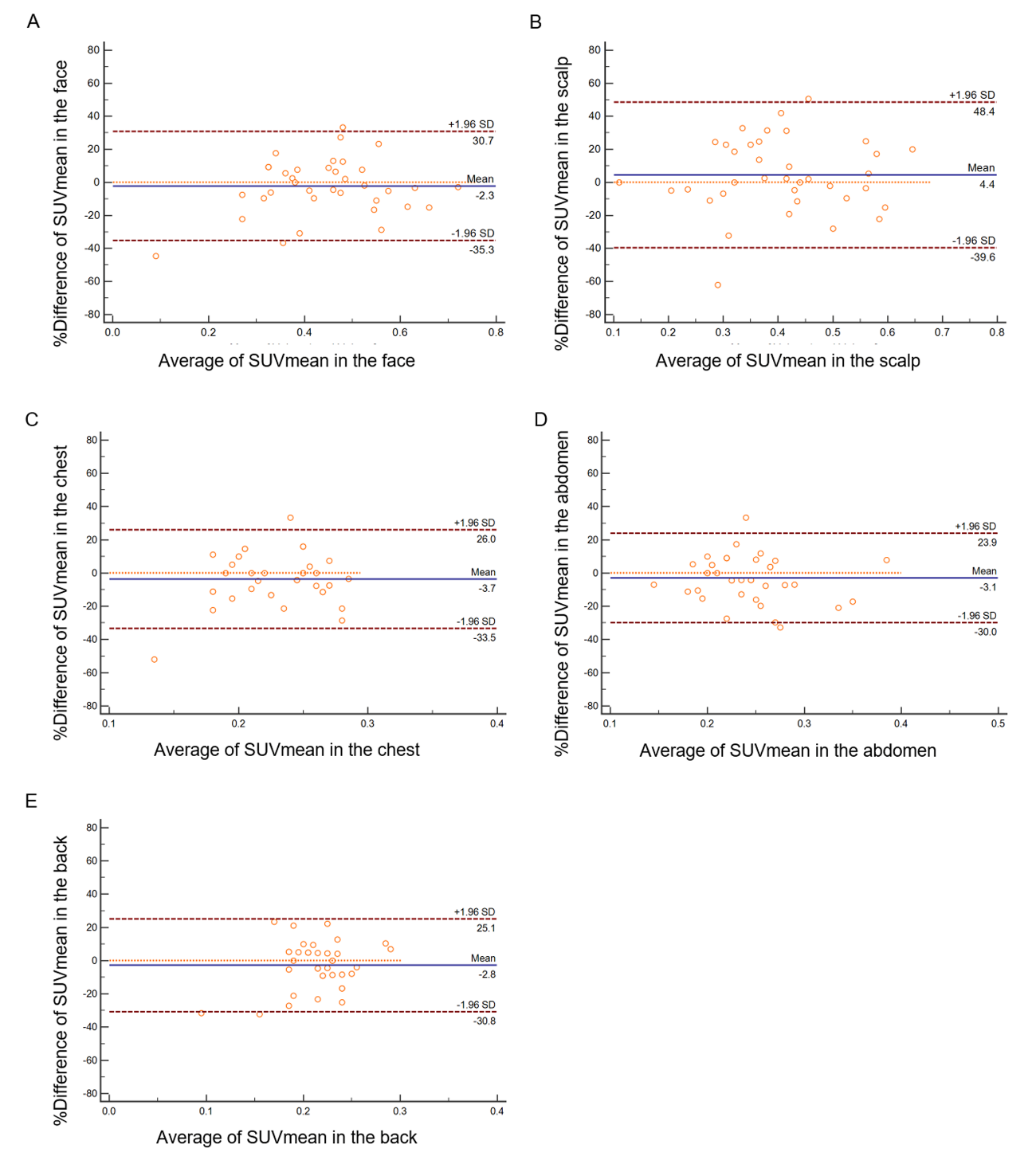


**S1 Fig.** Bland Altman plots for difference of SUVmean between the repeated examinations in face (A), scalp (B), chest (C), abdomen (D), and back (E) regions.
