# Supplementary material for: Physiological skin FDG uptake: A quantitative and regional distribution assessment using PET/MRI
Source: PLoS One. 2021 Mar 26;16(3):e0249304. doi: 10.1371/journal.pone.0249304 (PMC7997016; doi:10.1371/journal.pone.0249304)
Supplement: S3 Fig — (DOCX) [file pone.0249304.s003.docx]

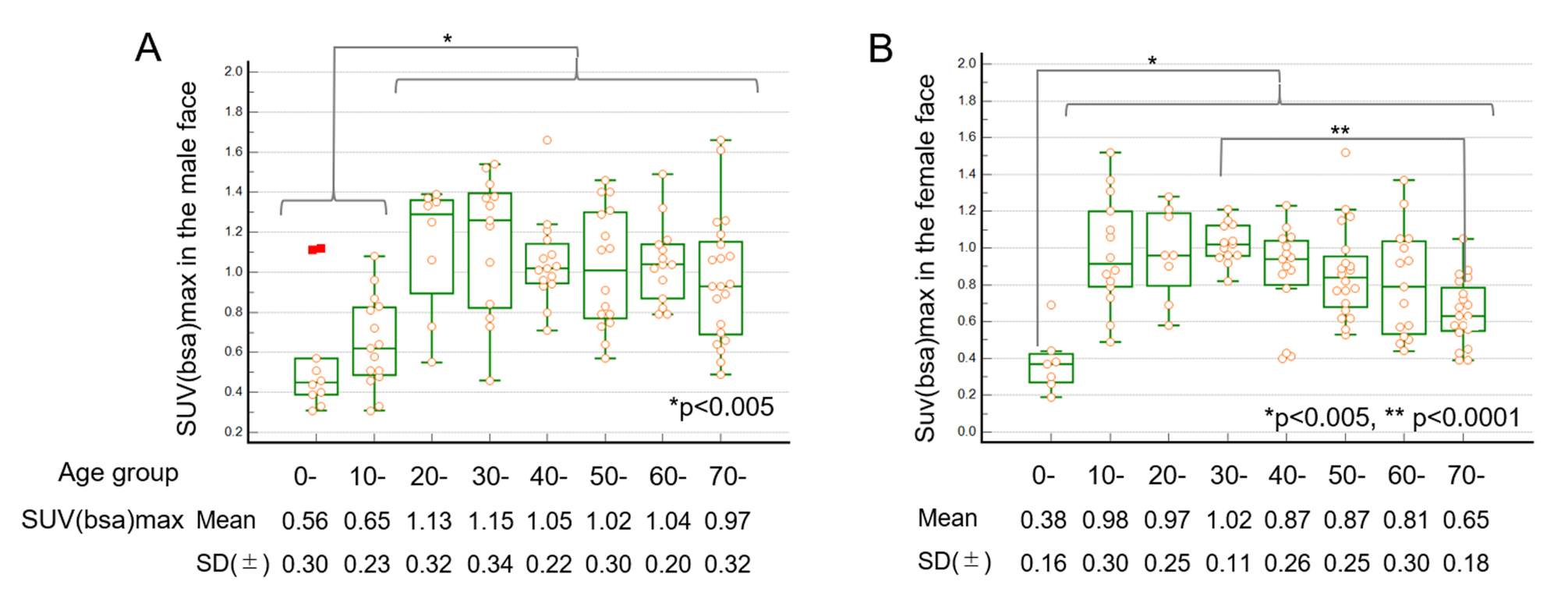


**S3 Fig.** **Relationship between skin SUV(bsa)max in the face and age group.** The box and whisker plot representing the skin SUVmax normalized by body-surface area in male (n=112) (A) and female (n=112) (B) patients.
