# Supplementary material for: Physiological skin FDG uptake: A quantitative and regional distribution assessment using PET/MRI
Source: PLoS One. 2021 Mar 26;16(3):e0249304. doi: 10.1371/journal.pone.0249304 (PMC7997016; doi:10.1371/journal.pone.0249304)
Supplement: S1 Table — (DOCX) [file pone.0249304.s005.docx]

**S1 Table.** Repeatability of skin SUVmean in each region (n=37)

| Region | Mean difference [%] | Lower limits of agreements [%] | Upper limits of agreements [%] | Coefficient of repeatability |
| --- | --- | --- | --- | --- |
| Face | -2.3 (-7.9 to 3.3) | -35.2 (-45.0 to -25.6) | 30.7 (21.0 to 40.4) | 0.136 (0.111 to 0.176) |
| Scalp | 4.4 (-3.1 to 11.9) | -39.6 (-52.5 to -26.7) | 48.4 (35.5 to 61.3) | 0.178 (0.145 to 0.231) |
| Chest | -3.7 (-8.8 to 1.3) | -33.5 (-42.2 to -24.8) | 26.0 (17.3 to 34.7) | 0.064 (0.052 to 0.083) |
| Abdomen | -3.1 (-7.6 to 1.5) | -30.0 (-37.9 to -22.1) | 23.9 (16.0 to 31.8) | 0.070 (0.057 to 0.091) |
| Back | -2.8 (-7.6 to 1.9) | -30.8 (-39.0 to -22.6) | 25.1 (16.9 to 33.3) | 0.055 (0.045 to 0.071) |

Numbers in the parenthesis showed 95% confidence interval
