# Supplementary material for: Physiological skin FDG uptake: A quantitative and regional distribution assessment using PET/MRI
Source: PLoS One. 2021 Mar 26;16(3):e0249304. doi: 10.1371/journal.pone.0249304 (PMC7997016; doi:10.1371/journal.pone.0249304)
Supplement: S2 Table — (DOCX) [file pone.0249304.s006.docx]

**S2 Table.** SUVmax of each region (n=37)

| Region | Median (interquartile range) | Statistically different (p<0.05) from region # |
| --- | --- | --- |
| 1. Face | 3.14 (2.43 to 3.61) | 2), 3), 4), 5) |
| 1. Scalp | 2.01 (1.68 to 2.40) | 1), 3), 4), 5) |
| 1. Chest | 1.26 (1.12 to 1.55) | 1), 2) |
| 1. Abdomen | 1.14 (0.93 to 1.38) | 1), 2), 5) |
| 1. Back | 1.46 (1.21 to 1.77) | 1), 2), 4) |
