# Supplementary material for: Physiological skin FDG uptake: A quantitative and regional distribution assessment using PET/MRI
Source: PLoS One. 2021 Mar 26;16(3):e0249304. doi: 10.1371/journal.pone.0249304 (PMC7997016; doi:10.1371/journal.pone.0249304)
Supplement: S3 Table — (DOCX) [file pone.0249304.s007.docx]

**S3 Table.** SUVmax in the face in each sex and age group (n=224)

| Age group | n | Median (interquartile range) | Statistically different (p<0.05) from age group # | n | Median (interquartile range) | Statistically different (p<0.05) from age group # |  |
| --- | --- | --- | --- | --- | --- | --- | --- |
|  | Male (n=112) | | | Female (n=112) | | | |
| 1) 0-10 | 10 | 1.33 (0.94 to 1.36) | 3), 4), 5), 6), 7), 8) | 7 | 0.98 (0.73 to 1.06) | 2), 3), 4), 5), 6), 7), 8) |  |
| 2) 10-20 | 15 | 2.05 (1.29 to 2.94) | 3), 4), 5), 6), 7), 8) | 14 | 3/05 (2.25 to 2.90) | 1) |  |
| 3) 20-30 | 8 | 4.34 (3.25 to 5.35) | 1), 2) | 8 | 3.24 (2.77 to 4.13) | 1) |  |
| 4) 30-40 | 13 | 4.35 (3.29 to 5.31) | 1), 2) | 13 | 3.83 (3.40 to 4.05) | 1), 8) |  |
| 5) 40-50 | 15 | 3.79 (3.35 to 4.13) | 1), 2) | 15 | 3.26 (2.95 to 4.05) | 1) |  |
| 6) 50-60 | 16 | 3.68 (2.68 to 4.63) | 1), 2) | 20 | 3.04 (2.48 to 3.34) | 1) |  |
| 7) 60-70 | 14 | 3.89 (3.39 to 4.15) | 1), 2) | 15 | 2.77 (1.92 to 3.49) | 1) |  |
| 8) 70-80 | 21 | 3.87 (2.79 to 4.31) | 1), 2) | 20 | 2.27 (1.80 to 2.76) | 1), 4) |  |
