# Supplementary material for: Physiological skin FDG uptake: A quantitative and regional distribution assessment using PET/MRI
Source: PLoS One. 2021 Mar 26;16(3):e0249304. doi: 10.1371/journal.pone.0249304 (PMC7997016; doi:10.1371/journal.pone.0249304)
Supplement: S4 Table — (DOCX) [file pone.0249304.s008.docx]

**S4 Table.** Raw-data table of patients for repeatability assessment of SUV in whole skin and liver

| No | Age | 1st examination (n=37) | | | | | 2nd examination (n=37) | | | | |
| --- | --- | --- | --- | --- | --- | --- | --- | --- | --- | --- | --- |
|  |  | BMI | SUVmax | SUVmean | SUVmax | SUVmean | BMI | SUVmax | SUVmean | SUVmax | SUVmean |
|  |  |  | Whole skin | | Liver | |  | Whole skin | | Liver | |
| 1 | 82 | 15.5 | 1.87 | 0.23 | 2.52 | 2.04 | 16.8 | 1.90 | 0.28 | 2.58 | 1.94 |
| 2 | 61 | 20.0 | 6.80 | 0.25 | 3.24 | 2.60 | 19.9 | 6.28 | 0.24 | 3.67 | 2.65 |
| 3 | 60 | 25.7 | 2.96 | 0.19 | 2.63 | 1.91 | 26.0 | 3.41 | 0.21 | 2.77 | 2.01 |
| 4 | 49 | 22.8 | 3.92 | 0.23 | 2.56 | 1.79 | 23.0 | 3.68 | 0.20 | 1.93 | 1.38 |
| 5 | 66 | 22.3 | 3.57 | 0.25 | 2.70 | 2.23 | 22.0 | 3.12 | 0.23 | 2.56 | 2.07 |
| 6 | 78 | 28.4 | 4.14 | 0.29 | 3.50 | 2.87 | 23.1 | 4.25 | 0.30 | 3.32 | 2.37 |
| 7 | 67 | 19.9 | 2.89 | 0.24 | 2.62 | 2.14 | 20.2 | 2.92 | 0.24 | 2.90 | 2.30 |
| 8 | 10 | 12.9 | 0.92 | 0.10 | 1.43 | 1.13 | 12.7 | 0.92 | 0.12 | 1.88 | 1.52 |
| 9 | 70 | 19.2 | 1.99 | 0.21 | 2.88 | 2.23 | 18.5 | 2.06 | 0.20 | 2.70 | 2.14 |
| 10 | 53 | 21.7 | 2.53 | 0.21 | 2.45 | 1.97 | 21.5 | 3.19 | 0.18 | 2.89 | 2.22 |
| 11 | 60 | 27.6 | 3.61 | 0.18 | 2.94 | 2.03 | 30.0 | 2.74 | 0.22 | 3.49 | 2.64 |
| 12 | 65 | 22.8 | 4.07 | 0.28 | 3.41 | 2.49 | 23.1 | 3.68 | 0.28 | 3.33 | 2.50 |
| 13 | 59 | 18.9 | 4.88 | 0.25 | 2.52 | 2.00 | 18.7 | 5.44 | 0.31 | 3.57 | 2.66 |
| 14 | 79 | 17.0 | 4.90 | 0.27 | 2.86 | 2.41 | 18.8 | 3.67 | 0.24 | 2.98 | 2.35 |
| 15 | 50 | 23.4 | 1.98 | 0.27 | 2.88 | 2.33 | 22.2 | 2.01 | 0.24 | 2.45 | 1.95 |
| 16 | 47 | 21.9 | 1.88 | 0.24 | 3.06 | 2.42 | 20.3 | 2.16 | 0.20 | 2.13 | 1.52 |
| 17 | 64 | 21.6 | 4.35 | 0.24 | 3.92 | 3.14 | 21.4 | 3.72 | 0.22 | 3.26 | 2.69 |
| 18 | 63 | 18.4 | 2.93 | 0.23 | 2.18 | 1.67 | 15.6 | 3.14 | 0.24 | 2.57 | 2.08 |
| 19 | 63 | 17.4 | 3.10 | 0.20 | 2.59 | 2.02 | 18.3 | 3.02 | 0.19 | 2.60 | 2.05 |
| 20 | 33 | 23.5 | 3.91 | 0.25 | 3.14 | 2.41 | 21.6 | 3.49 | 0.30 | 2.87 | 2.28 |
| 21 | 84 | 27.1 | 3.18 | 0.24 | 2.78 | 2.05 | 26.5 | 3.29 | 0.23 | 3.12 | 2.30 |
| 22 | 77 | 20.7 | 2.34 | 0.22 | 2.89 | 2.26 | 20.3 | 2.27 | 0.21 | 2.87 | 2.23 |
| 23 | 84 | 22.3 | 2.99 | 0.24 | 2.64 | 2.05 | 21.7 | 3.2 | 0.24 | 2.96 | 2.35 |
| 24 | 80 | 20.9 | 2.14 | 0.24 | 2.66 | 2.02 | 18.6 | 2.8 | 0.27 | 2.43 | 1.87 |
| 25 | 61 | 26.0 | 3.46 | 0.28 | 3.08 | 2.28 | 26.6 | 4.16 | 0.30 | 2.96 | 2.20 |
| 26 | 72 | 27.1 | 2.46 | 0.26 | 3.18 | 2.48 | 27.4 | 2.3 | 0.24 | 2.93 | 2.40 |
| 27 | 66 | 22.1 | 3.16 | 0.23 | 3.12 | 2.61 | 22.4 | 2.87 | 0.24 | 3.45 | 2.86 |
| 28 | 85 | 33.8 | 3.69 | 0.25 | 4.16 | 3.07 | 31.6 | 4.37 | 0.25 | 3.75 | 3.16 |
| 29 | 76 | 22.4 | 3.35 | 0.16 | 3.41 | 2.54 | 22.5 | 3.33 | 0.21 | 3.45 | 2.79 |
| 30 | 67 | 17.9 | 1.64 | 0.23 | 2.06 | 1.46 | 17.7 | 1.79 | 0.25 | 2.29 | 1.78 |
| 31 | 47 | 21.3 | 3.22 | 0.27 | 2.89 | 2.28 | 22.0 | 3.4 | 0.27 | 2.72 | 2.09 |
| 32 | 51 | 27.5 | 2.68 | 0.24 | 3.28 | 2.45 | 29.0 | 2.85 | 0.24 | 3.24 | 2.57 |
| 33 | 76 | 14.6 | 2.61 | 0.26 | 2.62 | 1.99 | 18.4 | 3.2 | 0.21 | 3.11 | 2.34 |
| 34 | 12 | 22.3 | 3.14 | 0.27 | 2.50 | 1.81 | 22.1 | 2.56 | 0.25 | 2.21 | 1.59 |
| 35 | 67 | 23.1 | 3.62 | 0.23 | 3.80 | 2.76 | 22.6 | 3.86 | 0.23 | 3.99 | 2.93 |
| 36 | 58 | 21.6 | 3.55 | 0.22 | 2.87 | 2.12 | 21.4 | 3.76 | 0.24 | 2.99 | 2.29 |
| 37 | 62 | 24.4 | 2.84 | 0.25 | 3.67 | 2.92 | 21.2 | 2.34 | 0.25 | 3.53 | 2.85 |
