# Supplementary material for: Physiological skin FDG uptake: A quantitative and regional distribution assessment using PET/MRI
Source: PLoS One. 2021 Mar 26;16(3):e0249304. doi: 10.1371/journal.pone.0249304 (PMC7997016; doi:10.1371/journal.pone.0249304)
Supplement: S5 Table — (DOCX) [file pone.0249304.s009.docx]

**S5 Table.** Raw-data table of patients for relationship of skin and liver SUVmax with age

| Male (n=112) | | | | | | | Female (n=112) | | | | | | |
| --- | --- | --- | --- | --- | --- | --- | --- | --- | --- | --- | --- | --- | --- |
| No | Age | BMI | Skin  SUV(bw) max | Skin  SUV(lbm) max | Skin  SUV(bsa) max | Liver  SUV(bw) max | No | Age | BMI | Skin  SUV(bw) max | Skin  SUV(lbm) max | Skin  SUV(bsa) max | Liver SUV(bw)  max |
| 1 | 1 | 16.6 | 2.20 | 1.96 | 1.12 | 2.18 | 1 | 1 | 15.7 | 1.15 | 0.95 | 0.44 | 1.96 |
| 2 | 4 | 15.6 | 2.71 | 2.49 | 1.11 | 1.53 | 2 | 1 | 14.8 | 1.72 | 1.48 | 0.69 | 1.99 |
| 3 | 4 | 15.1 | 0.80 | 0.73 | 0.33 | 1.36 | 3 | 1 | 15.6 | 0.61 | 0.43 | 0.19 | 2.14 |
| 4 | 4 | 14.7 | 0.92 | 0.86 | 0.39 | 1.23 | 4 | 2 | 16.9 | 0.71 | 0.51 | 0.26 | 2.19 |
| 5 | 5 | 14.6 | 1.38 | 1.16 | 0.51 | 1.14 | 5 | 3 | 13.6 | 0.97 | 0.79 | 0.38 | 1.09 |
| 6 | 5 | 14.3 | 1.05 | 0.98 | 0.44 | 1.46 | 6 | 6 | 15.2 | 0.74 | 0.63 | 0.30 | 1.20 |
| 7 | 6 | 13.8 | 1.30 | 1.26 | 0.57 | 1.16 | 7 | 7 | 15.3 | 0.95 | 0.80 | 0.37 | 1.70 |
| 8 | 8 | 14.6 | 1.20 | 1.11 | 0.46 | 2.51 | 8 | 11 | 15.0 | 1.97 | 1.66 | 0.73 | 2.14 |
| 9 | 8 | 13.5 | 0.76 | 0.71 | 0.31 | 2.10 | 9 | 11 | 18.2 | 2.72 | 2.18 | 0.86 | 2.95 |
| 10 | 9 | 13.3 | 0.99 | 0.93 | 0.40 | 1.98 | 10 | 12 | 10.9 | 2.20 | 1.79 | 0.88 | 1.27 |
| 11 | 10 | 13.1 | 0.78 | 0.74 | 0.31 | 1.31 | 11 | 12 | 11.4 | 2.43 | 2.20 | 1.06 | 1.51 |
| 12 | 10 | 14.2 | 1.52 | 1.41 | 0.58 | 1.43 | 12 | 12 | 12.3 | 2.90 | 2.58 | 1.20 | 1.48 |
| 13 | 10 | 12.9 | 1.53 | 1.45 | 0.64 | 2.43 | 13 | 12 | 19.2 | 1.61 | 1.27 | 0.49 | 2.71 |
| 14 | 10 | 12.7 | 1.21 | 1.14 | 0.51 | 1.86 | 14 | 13 | 13.2 | 2.38 | 2.08 | 0.95 | 1.43 |
| 15 | 10 | 13.5 | 0.80 | 0.75 | 0.33 | 1.58 | 15 | 13 | 13.5 | 2.81 | 2.44 | 1.10 | 1.88 |
| 16 | 10 | 14.5 | 1.17 | 1.08 | 0.46 | 1.60 | 16 | 14 | 22.7 | 2.06 | 1.51 | 0.58 | 2.47 |
| 17 | 12 | 24.2 | 4.03 | 3.27 | 1.08 | 2.15 | 17 | 16 | 23.1 | 2.88 | 2.10 | 0.79 | 2.16 |
| 18 | 12 | 20.1 | 1.68 | 1.45 | 0.51 | 1.92 | 18 | 16 | 19.9 | 2.63 | 2.05 | 0.82 | 2.77 |
| 19 | 12 | 22.4 | 3.03 | 2.53 | 0.83 | 2.36 | 19 | 17 | 25.0 | 5.09 | 3.58 | 1.31 | 3.11 |
| 20 | 12 | 24.8 | 3.09 | 2.48 | 0.81 | 2.29 | 20 | 18 | 24.0 | 5.21 | 3.72 | 1.37 | 2.74 |
| 21 | 12 | 22.3 | 3.14 | 2.61 | 0.87 | 2.65 | 21 | 18 | 23.6 | 5.74 | 4.14 | 1.52 | 2.86 |
| 22 | 13 | 22.1 | 2.62 | 2.18 | 0.72 | 2.35 | 22 | 20 | 21.4 | 4.16 | 3.14 | 1.21 | 2.39 |
| 23 | 14 | 15.2 | 1.36 | 1.25 | 0.48 | 1.52 | 23 | 20 | 20.2 | 2.98 | 2.30 | 0.90 | 2.80 |
| 24 | 14 | 17.8 | 1.95 | 1.73 | 0.62 | 1.75 | 24 | 24 | 19.0 | 3.04 | 2.40 | 0.96 | 2.37 |
| 25 | 15 | 15.9 | 2.85 | 2.59 | 0.96 | 1.62 | 25 | 25 | 19.3 | 1.91 | 1.50 | 0.58 | 2.75 |
| 26 | 20 | 20.5 | 4.78 | 4.08 | 1.35 | 3.41 | 26 | 26 | 18.9 | 4.12 | 2.01 | 1.28 | 2.55 |
| 27 | 21 | 27.6 | 5.73 | 4.41 | 1.33 | 3.27 | 27 | 27 | 22.5 | 3.40 | 2.51 | 0.96 | 3.06 |
| 28 | 23 | 18.2 | 3.50 | 3.06 | 1.06 | 2.54 | 28 | 27 | 22.8 | 4.19 | 3.10 | 1.17 | 2.91 |
| 29 | 23 | 23.7 | 4.85 | 3.95 | 1.25 | 3.98 | 29 | 28 | 16.4 | 2.12 | 1.75 | 0.69 | 3.18 |
| 30 | 24 | 27.3 | 5.72 | 4.42 | 1.39 | 2.93 | 30 | 31 | 48.8 | 5.88 | 2.03 | 1.04 | 5.30 |
| 31 | 26 | 27.5 | 2.40 | 1.80 | 0.55 | 3.55 | 31 | 33 | 23.5 | 3.91 | 2.82 | 1.02 | 3.27 |
| 32 | 28 | 19.4 | 2.49 | 2.16 | 0.73 | 3.17 | 32 | 34 | 29.1 | 4.78 | 3.05 | 1.13 | 3.33 |
| 33 | 29 | 21.8 | 5.23 | 4.37 | 1.37 | 3.58 | 33 | 34 | 18.1 | 2.67 | 2.14 | 0.82 | 2.66 |
| 34 | 31 | 28.4 | 3.12 | 2.37 | 0.73 | 3.41 | 34 | 34 | 21.6 | 3.49 | 2.62 | 0.96 | 3.08 |
| 35 | 31 | 28.8 | 3.29 | 2.49 | 0.77 | 2.87 | 35 | 36 | 20.7 | 4.05 | 3.10 | 1.15 | 2.95 |
| 36 | 32 | 24.0 | 3.21 | 2.61 | 0.84 | 4.06 | 36 | 36 | 18.0 | 2.98 | 2.40 | 0.95 | 2.67 |
| 37 | 32 | 19.2 | 4.68 | 4.07 | 1.38 | 3.60 | 37 | 37 | 19.0 | 3.94 | 3.11 | 1.21 | 2.81 |
| 38 | 34 | 28.8 | 5.81 | 4.39 | 1.37 | 3.67 | 38 | 37 | 20.4 | 3.40 | 2.62 | 1.00 | 2.52 |
| 39 | 34 | 31.0 | 5.80 | 3.53 | 1.33 | 3.72 | 39 | 37 | 25.0 | 3.50 | 2.45 | 0.92 | 3.84 |
| 40 | 36 | 20.4 | 4.51 | 3.86 | 1.26 | 3.08 | 40 | 37 | 18.7 | 3.35 | 2.65 | 1.03 | 2.61 |
| 41 | 37 | 26.6 | 4.97 | 3.88 | 1.23 | 2.66 | 41 | 37 | 23.2 | 3.60 | 2.62 | 0.96 | 3.71 |
| 42 | 37 | 19.8 | 5.02 | 4.33 | 1.44 | 2.66 | 42 | 39 | 25.1 | 4.20 | 2.94 | 1.12 | 2.66 |
| 43 | 38 | 19.5 | 5.31 | 4.61 | 1.52 | 2.34 | 43 | 41 | 39.0 | 5.05 | 2.50 | 1.01 | 3.85 |
| 44 | 38 | 23.0 | 5.73 | 4.71 | 1.54 | 3.12 | 44 | 41 | 21.4 | 3.28 | 2.49 | 0.95 | 2.39 |
| 45 | 38 | 17.1 | 3.31 | 2.96 | 1.05 | 2.55 | 45 | 42 | 31.6 | 4.57 | 2.74 | 1.05 | 3.93 |
| 46 | 39 | 23.8 | 1.74 | 1.42 | 0.46 | 2.42 | 46 | 44 | 19.9 | 3.10 | 2.40 | 0.94 | 2.77 |
| 47 | 41 | 17.8 | 2.29 | 2.03 | 0.71 | 2.36 | 47 | 45 | 25.3 | 1.59 | 1.11 | 0.41 | 4.29 |
| 48 | 41 | 19.0 | 3.59 | 3.13 | 1.09 | 3.00 | 48 | 46 | 36.3 | 4.30 | 2.29 | 0.90 | 4.33 |
| 49 | 42 | 22.6 | 3.51 | 2.90 | 0.96 | 2.60 | 49 | 46 | 20.5 | 4.18 | 3.21 | 1.23 | 2.69 |
| 50 | 42 | 19.3 | 3.22 | 2.80 | 0.94 | 2.65 | 50 | 47 | 21.3 | 3.02 | 2.27 | 0.86 | 2.89 |
| 51 | 44 | 28.1 | 5.04 | 3.84 | 1.21 | 3.32 | 51 | 47 | 21.9 | 1.54 | 1.15 | 0.43 | 3.05 |
| 52 | 45 | 22.4 | 2.86 | 2.38 | 0.80 | 1.85 | 52 | 47 | 20.3 | 1.38 | 1.06 | 0.40 | 2.21 |
| 53 | 45 | 20.6 | 3.69 | 3.15 | 1.03 | 2.00 | 53 | 47 | 22.0 | 3.14 | 2.33 | 0.88 | 2.80 |
| 54 | 46 | 31.3 | 4.54 | 3.30 | 1.01 | 3.53 | 54 | 49 | 18.9 | 3.11 | 2.45 | 0.97 | 2.55 |
| 55 | 48 | 23.3 | 3.77 | 3.09 | 1.02 | 3.38 | 55 | 49 | 22.8 | 3.92 | 2.88 | 1.06 | 2.32 |
| 56 | 48 | 19.2 | 3.16 | 2.75 | 0.93 | 2.66 | 56 | 49 | 23.0 | 2.88 | 2.11 | 0.78 | 1.99 |
| 57 | 48 | 17.6 | 3.48 | 3.09 | 1.07 | 2.42 | 57 | 49 | 19.5 | 3.77 | 2.95 | 1.11 | 2.31 |
| 58 | 48 | 22.4 | 3.58 | 2.98 | 0.98 | 2.55 | 58 | 50 | 24.0 | 2.96 | 2.12 | 0.78 | 2.68 |
| 59 | 48 | 19.7 | 5.82 | 5.02 | 1.66 | 3.58 | 59 | 50 | 22.2 | 1.86 | 1.38 | 0.53 | 2.52 |
| 60 | 49 | 18.8 | 4.26 | 3.72 | 1.24 | 2.46 | 60 | 51 | 27.5 | 3.04 | 2.01 | 0.77 | 3.29 |
| 61 | 49 | 20.2 | 4.00 | 3.43 | 1.16 | 3.17 | 61 | 52 | 28.0 | 4.06 | 2.67 | 0.99 | 2.96 |
| 62 | 51 | 29.0 | 5.15 | 3.86 | 1.18 | 4.33 | 62 | 53 | 22.6 | 3.10 | 2.28 | 0.86 | 2.75 |
| 63 | 52 | 22.0 | 2.71 | 2.26 | 0.73 | 3.38 | 63 | 53 | 21.7 | 2.46 | 1.84 | 0.70 | 2.56 |
| 64 | 52 | 23.6 | 4.24 | 3.47 | 1.12 | 2.53 | 64 | 53 | 22.4 | 3.12 | 2.30 | 0.88 | 3.10 |
| 65 | 53 | 22.2 | 5.15 | 4.30 | 1.40 | 2.89 | 65 | 53 | 18.0 | 2.83 | 2.28 | 0.90 | 3.20 |
| 66 | 54 | 20.7 | 2.86 | 2.44 | 0.79 | 3.47 | 66 | 54 | 23.0 | 4.38 | 3.20 | 1.21 | 3.39 |
| 67 | 55 | 19.1 | 2.58 | 2.25 | 0.75 | 2.78 | 67 | 54 | 26.9 | 2.59 | 1.74 | 0.66 | 3.54 |
| 68 | 55 | 22.5 | 5.17 | 4.30 | 1.40 | 4.16 | 68 | 54 | 19.1 | 4.96 | 3.91 | 1.52 | 3.41 |
| 69 | 56 | 16.2 | 4.48 | 4.06 | 1.46 | 2.46 | 69 | 55 | 20.6 | 3.99 | 3.05 | 1.17 | 2.88 |
| 70 | 56 | 23.4 | 4.94 | 4.05 | 1.31 | 2.84 | 70 | 56 | 22.2 | 2.20 | 1.63 | 0.62 | 3.03 |
| 71 | 57 | 15.2 | 2.34 | 2.15 | 0.79 | 2.40 | 71 | 57 | 20.6 | 3.05 | 2.33 | 0.89 | 3.59 |
| 72 | 57 | 23.9 | 4.31 | 3.51 | 1.11 | 3.13 | 72 | 57 | 19.1 | 2.95 | 2.33 | 0.92 | 2.45 |
| 73 | 57 | 23.4 | 2.18 | 1.78 | 0.57 | 2.57 | 73 | 57 | 25.8 | 2.40 | 1.65 | 0.62 | 2.55 |
| 74 | 58 | 18.4 | 2.99 | 2.63 | 0.91 | 2.22 | 74 | 57 | 17.1 | 2.53 | 2.06 | 0.82 | 2.68 |
| 75 | 58 | 21.7 | 2.36 | 1.98 | 0.64 | 2.79 | 75 | 58 | 19.9 | 1.90 | 1.47 | 0.56 | 2.96 |
| 76 | 59 | 19.3 | 2.89 | 2.51 | 0.83 | 2.68 | 76 | 58 | 20.9 | 3.99 | 3.04 | 1.15 | 2.80 |
| 77 | 59 | 20.7 | 4.52 | 3.84 | 1.29 | 2.93 | 77 | 59 | 19.2 | 2.49 | 1.95 | 0.77 | 2.41 |
| 78 | 60 | 19.1 | 3.84 | 3.34 | 1.14 | 3.15 | 78 | 60 | 26.0 | 2.03 | 1.39 | 0.52 | 2.64 |
| 79 | 61 | 26.0 | 3.46 | 2.72 | 0.87 | 3.08 | 79 | 61 | 20.0 | 2.27 | 1.84 | 0.70 | 3.44 |
| 80 | 61 | 22.3 | 5.52 | 4.59 | 1.49 | 3.33 | 80 | 62 | 24.4 | 3.84 | 2.72 | 1.05 | 3.91 |
| 81 | 62 | 26.6 | 4.67 | 3.65 | 1.16 | 3.74 | 81 | 62 | 21.2 | 2.68 | 2.03 | 0.79 | 3.53 |
| 82 | 64 | 16.2 | 3.37 | 3.05 | 1.11 | 2.64 | 82 | 63 | 17.4 | 1.59 | 1.29 | 0.50 | 2.46 |
| 83 | 65 | 22.8 | 4.07 | 3.36 | 1.07 | 3.53 | 83 | 63 | 18.3 | 1.55 | 1.24 | 0.48 | 2.66 |
| 84 | 65 | 23.1 | 3.68 | 3.03 | 0.96 | 3.41 | 84 | 64 | 21.6 | 4.74 | 3.55 | 1.37 | 3.60 |
| 85 | 65 | 20.9 | 3.69 | 3.14 | 1.04 | 2.81 | 85 | 64 | 21.4 | 4.26 | 3.20 | 1.24 | 3.41 |
| 86 | 67 | 26.0 | 4.18 | 3.30 | 1.02 | 2.44 | 86 | 66 | 22.3 | 3.57 | 2.65 | 1.05 | 2.77 |
| 87 | 67 | 26.5 | 5.37 | 4.21 | 1.32 | 4.32 | 87 | 66 | 22.0 | 3.40 | 2.54 | 1.00 | 2.77 |
| 88 | 68 | 21.4 | 2.84 | 2.39 | 0.79 | 3.25 | 88 | 66 | 22.4 | 3.38 | 2.50 | 0.93 | 3.77 |
| 89 | 68 | 23.0 | 2.98 | 2.45 | 0.82 | 3.12 | 89 | 67 | 19.9 | 3.00 | 2.32 | 0.92 | 2.74 |
| 90 | 69 | 20.8 | 2.73 | 2.32 | 0.79 | 2.44 | 90 | 67 | 17.9 | 1.37 | 1.11 | 0.44 | 1.77 |
| 91 | 69 | 23.9 | 3.99 | 3.24 | 1.04 | 3.70 | 91 | 68 | 17.7 | 1.81 | 1.46 | 0.58 | 2.21 |
| 92 | 70 | 22.2 | 6.12 | 5.10 | 1.66 | 2.78 | 92 | 68 | 22.6 | 2.05 | 1.51 | 0.57 | 4.18 |
| 93 | 70 | 27.7 | 5.02 | 3.86 | 1.19 | 3.76 | 93 | 70 | 19.2 | 1.48 | 1.17 | 0.45 | 2.93 |
| 94 | 71 | 26.5 | 4.21 | 3.30 | 1.06 | 3.19 | 94 | 70 | 18.5 | 1.86 | 1.39 | 0.54 | 2.85 |
| 95 | 72 | 18.9 | 2.15 | 1.72 | 0.55 | 3.21 | 95 | 72 | 27.1 | 2.46 | 1.65 | 0.63 | 3.40 |
| 96 | 74 | 22.8 | 2.79 | 2.31 | 0.74 | 3.38 | 96 | 72 | 27.4 | 2.30 | 1.53 | 0.59 | 3.18 |
| 97 | 74 | 22.7 | 3.15 | 2.62 | 0.87 | 2.66 | 97 | 73 | 22.4 | 3.14 | 2.32 | 0.88 | 3.37 |
| 98 | 75 | 24.1 | 6.14 | 4.98 | 1.61 | 3.64 | 98 | 73 | 14.6 | 1.62 | 1.38 | 0.58 | 2.67 |
| 99 | 75 | 31.2 | 5.46 | 3.96 | 1.25 | 3.64 | 99 | 73 | 18.4 | 2.01 | 1.60 | 0.63 | 2.65 |
| 100 | 75 | 20.5 | 3.29 | 2.68 | 0.89 | 3.20 | 100 | 74 | 20.7 | 1.94 | 1.48 | 0.58 | 2.96 |
| 101 | 75 | 19.5 | 4.31 | 3.73 | 1.26 | 2.86 | 101 | 74 | 20.3 | 2.27 | 1.74 | 0.69 | 3.16 |
| 102 | 75 | 21.7 | 4.06 | 3.41 | 1.14 | 2.96 | 102 | 74 | 22.5 | 2.57 | 1.89 | 0.72 | 3.80 |
| 103 | 76 | 28.4 | 2.97 | 2.25 | 0.70 | 3.56 | 103 | 75 | 17.0 | 3.19 | 2.60 | 1.05 | 3.29 |
| 104 | 76 | 23.1 | 1.81 | 1.49 | 0.49 | 3.70 | 104 | 76 | 18.8 | 2.69 | 2.13 | 0.84 | 3.03 |
| 105 | 77 | 21.2 | 3.76 | 3.18 | 1.08 | 2.69 | 105 | 76 | 15.5 | 1.11 | 0.93 | 0.39 | 2.50 |
| 106 | 77 | 25.3 | 2.40 | 1.91 | 0.61 | 2.59 | 106 | 76 | 16.8 | 1.29 | 1.06 | 0.43 | 2.44 |
| 107 | 78 | 20.9 | 2.19 | 1.86 | 0.64 | 2.53 | 107 | 77 | 16.6 | 1.14 | 0.96 | 0.39 | 2.85 |
| 108 | 78 | 22.2 | 3.40 | 2.84 | 0.93 | 3.43 | 108 | 77 | 27.1 | 3.18 | 2.13 | 0.82 | 2.81 |
| 109 | 78 | 18.6 | 2.11 | 1.85 | 0.66 | 2.49 | 109 | 77 | 22.3 | 2.58 | 1.91 | 0.75 | 2.88 |
| 110 | 79 | 27.3 | 8.27 | 3.48 | 1.07 | 3.24 | 110 | 79 | 26.5 | 3.29 | 2.23 | 0.86 | 3.15 |
| 111 | 79 | 25.0 | 3.56 | 2.85 | 0.93 | 3.00 | 111 | 79 | 33.8 | 2.98 | 1.70 | 0.68 | 4.16 |
| 112 | 79 | 30.6 | 4.13 | 3.03 | 0.94 | 4.45 | 112 | 79 | 31.6 | 2.39 | 1.44 | 0.56 | 4.13 |
