# Supplementary material for: Physiological skin FDG uptake: A quantitative and regional distribution assessment using PET/MRI
Source: PLoS One. 2021 Mar 26;16(3):e0249304. doi: 10.1371/journal.pone.0249304 (PMC7997016; doi:10.1371/journal.pone.0249304)
Supplement: Attachment — Submitted filename: PLoS_One_R1_Response_to_reviewers_v2.docx [file pone.0249304.s010.docx]

**Response to Reviewers**

Manuscript#: PONE‐D‐20‐29259

Title: Physiological skin FDG uptake: A quantitative and regional distribution assessment using PET/MRI

We thank the reviewers for the evaluation of our study and the editor for giving us the chance of resubmitting our manuscript. We are happy to address all comments point by point. We have made revisions that have been marked in colored fonts for ease of identification in the revised version of our manuscript.

**Reviewer #1:**

1. **Comments:** 298 exams altogether – I believe, although because of the convoluted description on it is hard to be certain.

**Response:** We apologize for any confusion our description may have caused. As you mentioned, the total number of assessed examinations was 298. The heading of Table 1 was modified to clearly show the numbers of assessed examinations per category.

2. **Comments:** I do somewhat question on the decision to use 2‐voxel thick VOIs for the <10‐year olds but 3‐voxel thick sections for the rest. …this bias would be more predictable and perhaps even compensated for using a partial volume correction based upon an age dependent function of skin thickness.

**Response:** Thank you for your suggestions. As you pointed out, differences in VOI thickness were due to age-dependent differences in skin thickness. The 9.375 mm thickness of 3 voxels was too large for the segmentation of the thin skin in children, especially when VOIs were adjacent to organs such as the brain with high physiological uptake. This resulted in a lot of manual adjustments to exclude voxels outside of the skin structure (as shown in the following figure).

However, we agree that this difference in the definition of VOIs may lead to unnecessary bias in this study. Therefore, we have performed additional image segmentation using the same VOI thickness (3 voxels) for all examinations. Accordingly, more manual adjustments were necessary for patients under the age of 10 years; however, the statistical analysis showed no difference in the results from the previous assessment with different VOI segmentations. The modified number of the manual adjustments are shown in the results section (Page 11, lines 174 to 176). A slight difference in SUVmax in the face was found in patients under 10 years of age, but the statistical results were not different in comparison to the previous assessment (Figure 6).

3. **Comments:** I assume the SUV metric used here is the one normalized by patient body‐weight (the formula is not described but my concern would apply equally had SUV normalized by lean‐body mass or body surface‐area etc. been used) and this is by no means a perfect normalizer. It is quite possible (likely even) that much of the FDG uptake age dependence described is actually due to the choice of normalizer. As a check, it would be interesting to see what the liver uptakes dependence on age was.

**Response:** Thank you very much for your extremely valuable comments. The SUV calculation was normalized to the patients’ body weight, as you mentioned. We added a description regarding the definition of the SUV calculation to the Image analysis section (Page 10, line 151). Furthermore, we do agree that the selection of a normalizer (body weight [bw], lean body mass [lbm], and body surface area [bsa]) could have an impact on the statistical difference in SUVs among age groups. Therefore, we calculated SUVmax with lbm and bsa as well to assess differences in SUVs among age groups and found that both SUV(lbm)max and SUV(bsa)max showed no difference in relation to age compared to SUV(bw)max, suggesting the age-dependency of the skin uptake was not due to the choice of the normalizer. Descriptions regarding the normalizers for SUV assessments were added to the discussion section (Page 16, line 261 to 269). The additional results for SUV(lbm)max and SUV(bsa)max are shown in S2 and S3 Figs. The relationship between liver FDG uptake and age has already been shown in the literature (Clin Nucl Med. 2013,38(6):422-5; Clin Imaging. 2010,34(5):348-50), but we checked the relationship of liver SUV(bw)max, SUV(lbm)max, and SUV(bsa)max with the patients’ age also in our study population. Similar to the results of the skin values, liver SUVmax values were significantly lower in the age group 0–9 years compared to those in adults. The age-related increase in SUVmax was, however, more gradual in females than males, and the decrease in SUVmax found in the skin of older women was not seen in their corresponding liver values. The age-related tendency was not different among SUVmax normalizers, suggesting that the liver SUVmax calculated by different metrics did not affect the quantitative assessment of differences among age groups.

4. **Comments:** the authors simply made as much of the raw data available as possible (e.g. a table of heights, weights, ages, SUVmeans, SUVmaxes etc. for each subject) and using VOIs of the same thickness for all subjects.

**Response:** According to your kind suggestion, we added supplemental tables (S4 and S5 Tables) containing the raw data of patients for the assessment of repeatability, regional distribution, and relationship with age. The tables also include the patients’ age, BMI, and additional SUV metrics (lbs and bsa).

5. **Comments:** the PET camera used contain silicon photomultipliers, but there’s no clear reason to think that these per se had any real influence on this study.

**Response:** Indeed, the manuscript did not assess or even discuss the contribution of silicon photomultipliers to the accuracy in skin uptake measurements. Therefore, we deleted the term “silicon multiplier” and “SiPM” from the manuscript.

**Reviewer #2:**

1. **Comments:** Discuss the possibility that the observed results are due to backscatter from bony surfaces in face, skull, sternum (not present in abdomen, etc.).

**Response:** Thank you very much for your comment. PET attenuation correction on PET/MRI is performed by MR-based pseudo-CT, which does not induce CT-derived artifacts such as beam-hardening artifacts. In the attenuation correction (AC) map (μ map) generated from MR-based pseudo-CT, only four-tissue segmentations (air, fat, water, and soft-tissue) were utilized for the correction without bone components except for the skull (CT-atlas or MR-based bone segmentation) (J Nucl Med 2012; 53:796–804). As you mentioned, CT-AC does have a “backscatter” phenomenon close to the bones, but MR-AC does not induce this artifact due to the lack of a bone component. We added descriptions regarding this issue (Page 15, line 257 to page 16, line 260).

2. **Comments:** Discuss the possibility that the observed results may be in part explained by the lack of self shielding in the higher noted uptake areas.

**Response:** The issue you comment on is important and is carefully addressed in the methods section. High uptakes by organs other than the skin including the brain, salivary glands, muscles, liver, and urinary system were excluded from the analysis, as described in the Image analysis section (Page 9, line 143 to page 10, line 147). As a result, manual adjustments were necessary for 69 of the 298 examinations. Nevertheless, a “spillover” effect as mentioned by the reviewer cannot be completely resolved and is described as a limitation of the study.

3. **Comments:** Discuss the possibility that the observed results may be the result of uptake in the blood pool (higher capillary blood at the surface in the noted higher uptake regions), in the eyes themselves (or salivary glands), in the nasopharangeal regions.

**Response:** Thank you for your valuable comments. We agree that the blood pool could be a possible reason for skin uptake. The SUVmax in the face, especially in the nose, was higher than that in the large vessels or mucosa of the nose (data not shown), and even higher than that in the blood-rich organ liver in 58% (131 of 224 examinations) of the patients (S5 Table) in our study. We consider that these results suggest that the skin uptake cannot be explained by the blood pool alone. The descriptions regarding the abovementioned results were added to the discussion section (Page 16, line 275 to page 17, line 279). As for the physiological uptakes of the orbit (external ocular muscle and lachrymal gland) and the tonsilla or salivary gland in the nasopharyngeal areas, we carefully exclude all those uptakes from the skin VOIs as shown in the Image analysis section. We add descriptions regarding those regions to the Image analysis section　(Page 10, line 145).

In addition to the revision of the manuscript according to the reviewers’ valuable comments, we corrected minor typographical errors as follows:

Table 1 (Page 6): Heading for the table.

Table 1 (Page 6): The interquartile range of the BMI of 1st examination for assessment of repeatability and regional distribution.

Table 1 (Page 6): The mean value of the BMI of female patients in 0-9 yrs. old for assessment for relationship with age.

Table 1 (Page 6): The interquartile range of the BMI of female patients in 0-9 yrs. old and 70-19 yrs. old for assessment for relationship with age.

Page 10, line 162: The name of the statistical analysis (paired t-test > the Wilcoxon’s signed-rank test).

Page 12, line 187-188: The percent mean difference and lower and upper limits of agreements of the SUVmax (and 95% CI) in the overall skin region.

Page 12, line 193-194: The coefficient of repeatability for the SUVmax (95% CI) in the overall skin region.

Page 14, line 227: The SUVmax of the face in female patients.
