# Supplementary material for: Physiological skin FDG uptake: A quantitative and regional distribution assessment using PET/MRI
Source: PLoS One. 2021 Mar 26;16(3):e0249304. doi: 10.1371/journal.pone.0249304 (PMC7997016; doi:10.1371/journal.pone.0249304)
Supplement: Attachment — Submitted filename: PLoS_One_R2_Response_to_reviewers_v2.docx [file pone.0249304.s011.docx]

**Response to Reviewers**

Manuscript#: PONE‐D‐20‐29259-R2

Title: Physiological skin FDG uptake: A quantitative and regional distribution assessment using PET/MRI

We thank the reviewers once again for evaluating of our study, and the editor for giving us the opportunity to resubmit our revised manuscript. We are happy to address all comments in a point-by-point manner. The revisions have been marked in colored font for ease of identification in the revised version of our manuscript.

**Reviewer #1:**

1. **Comments:** While the manuscript is much improved by the additional data included within the revision, the similarity in the trends for the SUV body-weight values as a function of age between the liver and skin actually heighten my concern about statements and data within the manuscript that might be interpreted by the reader to conclude that there exists a trend related to absolute FDG uptake. Although it was good to include the additional types of SUV metric (lean body mass and body surface area), it is wrong to conclude that similarity in their trends as a function of age (for example) means that trend exists for absolute FDG uptake. It is quite possible that all of these body-habitus metrics are poor (i.e. biased) normalizers for pediatric subjects. And because of the ease with which this point can be missed, it is imperative that the authors make this distinction clear, including in the Abstract, which may well be as far as some readers get.

**Response:** Thank you very much for your valuable comments. We completely agree with your opinion that the measured SUV could be highly related to the biased normalizer for body habitus, especially in pediatrics. Indeed, the SUV in the liver was also lower in pediatrics than in adult patients of both sexes, which suggests, as you mentioned, that the difference in skin SUV in pediatrics was not related to absolute FDG uptake at their age, but rather the biased normalization procedure for SUV based on their body habitus. We also agree that this influence of body size on accurate measurement of tracer uptake is a significant drawback of the SUV, which has already been confirmed more than twenty years ago (J Nucl Med 1995; 36:1836-1839. SUV: Standard Uptake of Silly Useless Value?). According to the reviewer’s valuable comments and the literature showing the limitations of SUV due to differences in body size, we have revised the discussion section’s description of the relationship between SUV and age groups (Page 16, lines 262 to 276). In addition, the conclusion sections in the Abstract and the main text have been revised accordingly.

2. **Comments:** So, while it is acceptable to report results showing that SUV is correlated with age, the authors should take pains to note that these trends do not necessarily hold for “FDG uptake” because SUV has not been definitively shown to correlate with FDG uptake across this patient population. If I am mistaken about this, the authors should of course make the case and cite the evidence that refutes my position, but otherwise all language suggesting that SUV is a quantitative measure of FDG uptake should be removed. As a case in point, the new language on page 16 line 261 in the Discussion, I believe is misleading. SUV is neither a robust nor particularly quantitative measure of FDG uptake, and to suggest that “biological and technical factors” that might be confounding that relationship have been ruled out, is simply incorrect.

**Response:** Thank you again for your valuable suggestions. We agree that the trends observed in the pediatrics in this study were not due to differences in FDG uptake, but rather differences in body size. Accordingly, we have removed the incorrect descriptions regarding “robustness” and “ruling out the biological and technical factors” from the discussion section (Page 16, line 262 and line 265).

In addition to the aforementioned revisions made according to the reviewers’ valuable comments, we have corrected a minor typographical error as follows:

Page 25, line 4: Figure legend for S4 Fig.

We thank the reviewers once again for their significant commitment in reviewing our manuscript and the kindness shown throughout this process.
